# Stabilizing Salt-Bridge Enhances Protein Thermostability by Reducing the Heat Capacity Change of Unfolding

**DOI:** 10.1371/journal.pone.0021624

**Published:** 2011-06-24

**Authors:** Chi-Ho Chan, Tsz-Ha Yu, Kam-Bo Wong

**Affiliations:** School of Life Sciences, Centre for Protein Science and Crystallography, The Chinese University of Hong Kong, Hong Kong, Shatin, Hong Kong SAR, China; National Institute for Medical Research, Medical Research Council, London, United Kingdom

## Abstract

Most thermophilic proteins tend to have more salt bridges, and achieve higher thermostability by up-shifting and broadening their protein stability curves. While the stabilizing effect of salt-bridge has been extensively studied, experimental data on how salt-bridge influences protein stability curves are scarce. Here, we used double mutant cycles to determine the temperature-dependency of the pair-wise interaction energy and the contribution of salt-bridges to ΔC_p_ in a thermophilic ribosomal protein L30e. Our results showed that the pair-wise interaction energies for the salt-bridges E6/R92 and E62/K46 were stabilizing and insensitive to temperature changes from 298 to 348 K. On the other hand, the pair-wise interaction energies between the control long-range ion-pair of E90/R92 were negligible. The ΔC_p_ of all single and double mutants were determined by Gibbs-Helmholtz and Kirchhoff analyses. We showed that the two stabilizing salt-bridges contributed to a reduction of ΔC_p_ by 0.8–1.0 kJ mol^−1^ K^−1^. Taken together, our results suggest that the extra salt-bridges found in thermophilic proteins enhance the thermostability of proteins by reducing ΔC_p_, leading to the up-shifting and broadening of the protein stability curves.

## Introduction

To survive in the hot habitats, proteins from thermophilic organisms are more thermal stable than their mesophilic homologs. The conformational stability of proteins is defined as the free energy difference between the native and the unfolded states, or the free energy of unfolding (ΔG_u_). ΔG_u_ varies with temperature as a curve function (i.e. the protein stability curve), which is described by the Gibbs-Helmholtz equation:
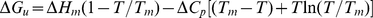
where T_m_ is the melting temperature, ΔH_m_ is the enthalpy change of protein unfolding at T_m_, and ΔC_p_ is the heat capacity change of unfolding.

Nojima and co-workers pointed out that protein thermostability, or increase in T_m_, can in theory be enhanced by: (i) up-shifting, (ii) broadening, and (iii) right-shifting of the protein stability curves [1]. Nussinov and co-workers studied the correlation between different thermodynamic parameters of 5 protein families and showed that thermophilic proteins prefer to increase T_m_ by up-shifting and broadening of their protein stability curves [2]. In a later study, Razvi and Scholtz systematically compared the protein stability curves of 26 thermo- and mesophilic homologous pairs of proteins. Regardless to the physical origins, they showed that over 70% of thermophilic proteins in their study achieve higher T_m_ by up-shifting and/or broadening of their protein stability curves as compared with their mesophilic homologous [3]. It is clear that most thermophilic proteins achieve higher thermostability by up-shifting and broadening of their protein stability curves.

The Gibbs-Helmholtz equation predicts that a smaller ΔC_p_ can up-shift and broaden a protein stability curve. For example, the curvature of the protein stability can be defined as the second derivative of the Gibbs-Helmholtz equation:
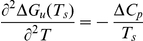
for T_s_ is the temperature where ΔG_u_ is maximum [2]. A reduction in ΔC_p_ will make the curvature less negative and, therefore, the protein stability curve is broadened. Similarly, it can be shown that a smaller ΔC_p_ can increase the maximum ΔG_u_: ΔG_u_(T_s_) = ΔH_m_−ΔC_p_(T_m_−T_s_), or in other words, the protein stability curve is up-shifted [2], provided that ΔH_m_ is increased or remains constant.

Consistent with the observation that most thermophilic proteins achieve higher thermostability by up-shifting and broadening of their protein stability curves, thermophilic proteins tend to have a much smaller value of ΔC_p_ than their mesophilic homologs [4]–[11]. For example, we have shown that the thermophilic ribosomal protein L30e from *Thermococcus celer* has a ΔC_p_ value of ∼5 kJ mol^−1^ K^−1^, which is much smaller than the value of ∼10 kJ mol^−1^ K^−1^ obtained for the mesophilic L30e from yeast [12].

In thermophilic proteins, one common strategy to enhance thermostability is to have more favorable surface charge-charge interactions. When compared with their mesophilic homologues, thermophilic proteins have more surface charged residues [13] and have an increased number of salt bridge [14]–[16]. The stabilizing role of the electrostatic interaction was first suggested by Perutz and Raidt based on their modeling studies [17], [18], and was experimentally verified by various strategies including optimization of surface charges [19], [20], removal of surface charges [21], [22], addition of new ion pairs [23], [24], and double mutant cycles [23], [25]–[34]. To study the contribution of charge-charge interactions to the thermostability and the reduced ΔC_p_ of thermophilic proteins, our group had systematically removed 26 surface charges on *T. celer* L30e by single charge-to-alanine substitutions. Most of the mutants results in decreases in T_m_ [21], indicated that the surface charges are mostly stabilizing in thermophilic protein. In another study, we showed that removal of favorable charge-charge interaction by single charge-to-neutral substitutions increases the ΔC_p_ value [12].

Here, we demonstrated that stabilizing salt-bridges enhance the thermostability of proteins by reducing the ΔC_p_. We used the double-mutant cycle to investigate the effect of pair-wise interaction of two salt bridges (E6/R92 and E62/K46) on protein thermostability and ΔC_p_. We showed that the two salt-bridges stabilized the *T. celer* L30e protein by ∼2–5 kJ mol^−1^, and the stabilizing effect was insensitive to temperature changes from 298–348 K. The contribution of the two salt-bridges to ΔC_p_ was determined independently by Gibbs-Helmholtz and Kirchhoff analyses. Our results showed that each salt-bridge contributed to a reduction of ΔC_p_ by 0.8–1.0 kJ mol^−1^ K^−1^. That salt-bridge reduces ΔC_p_ provides a structural basis for the large differences in ΔCp observed between thermophilic and mesophilic proteins.

## Results

### Design of variants

**Figure 1:**
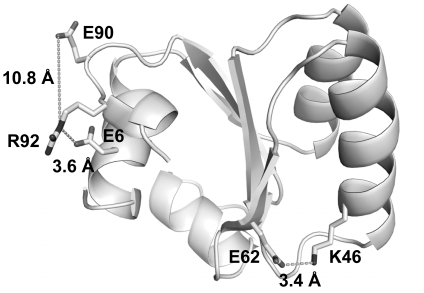
Design of L30e variants. The separation distances of the salt-bridges E6/R92 and E62/K46, and the control pair E90/R92 are indicated and represented by dashed lines.

In this study, we used the double-mutant cycle to investigate how salt-bridges contribute to the thermostability of proteins. We have selected two salt-bridges (E6/R92 and K46/E62), which are located on opposite sides of *T. celer* L30e (Figure 1). Charged residues were substituted with alanine. For Arg and Lys residues that have long side chain, substitutions to Met were made to mimic their long hydrophobic side chains. As a result, two double-mutant cycles were applied for each salt-bridge. As a negative control, we have also used double-mutant cycles to study the pair-wise interaction between E90 and R92, which have a long separation distance of 10.8 Å. A total of seven single mutants and six double mutants were generated (Table S1).

### Pair-wise Interaction energy between charge residues was determined by double-mutant cycles

Single charge-to-neutral substitutions suffer from the limitation that the residue being substituted may also form other interactions with the rest of the proteins. By canceling out these interactions using the double-mutant-cycle approach, one can estimate the contribution of the pair-wise interaction between the two oppositely charged residues in a salt-bridge [23]. The scheme presented in Figure S1 explains how the pair-wise interaction energy is determined by the double-mutant-cycle approach. For the theoretical background on the use of double-mutant cycle to determine the pair-wise interaction energy of salt-bridges, please refer to the work of Fersht and co-workers [23]. In brief, if pair-wise interaction exists between two oppositely charged residues, the ΔΔG_u_ for removing a negative charge from the wild-type protein (process A) should be smaller than that from M^−ve^ in which the positive charged residues has been substituted in prior (process B) (Figure S1). It is because in addition to the interaction made by the negative charge residue to the rest of the protein, the process A also removes the pair-wise interaction. Similar arguments could be applied to the ΔΔG for process C and D. We obtained the pair-wise interaction energy between the two charge residues (ΔΔG_int_) by: ΔΔG_int_ = [ΔG_u_(DM)−ΔG_u_(M^−ve^)]−[ΔG_u_(M^+ve^)−ΔG_u_ (WT)].

We have determined the free energy of unfolding (ΔG_u_) of the wild-type *T. celer* L30e and its variants by urea-induced denaturation at 298 K (Table 1), and calculated the values ΔΔG_int_ for the cycles E6A/R92A(M), E62A/K46A(M), and E90A/R92A(M) (Figure S1). The values of ΔΔG_int_ were in the range of 1.9–3.6 kJ mol^−1^ for the pairs of charged residues (E6/R92 and E62/K46) involved in salt-bridges. In contrast, the values of ΔΔG_int_ were close to zero for the control pairs (E90/R92). Taken together, our results suggest that the two salt-bridges of E6/R92 and E62/K46 contributed favorably to the stability of L30e.

**Table 1:** Free energy of unfolding (kJ mol^−1^) of *T. celer* L30e and its variants at 298–348 K.

Protein	298 K	308 K	318 K	328 K	338 K	348 K
Wild-type	34.9±0.5	35.0±0.5	32.4±0.5	30.8±0.4	26.6±0.4	20.9±0.5
E6A	27.5±0.3	27.7±0.3	25.5±0.3	23.3±0.3	19.0±0.3	14.4±0.3
K46A	29.8±0.3	29.7±0.4	26.7±0.4	24.2±0.3	19.0±0.3	13.3±0.4
K46M	31.1±0.3	30.6±0.4	28.3±0.3	26.3±0.4	22.4±0.3	17.4±0.2
E62A	28.5±0.3	28.7±0.3	25.7±0.3	23.3±0.3	18.0±0.2	12.1±0.3
E90A	32.7±0.4	32.7±0.4	29.8±0.5	29.0±0.4	24.2±0.4	19.8±1.5
R92A	33.9±0.5	33.6±0.5	31.3±0.6	30.8±0.5	25.0±0.4	19.1±0.3
R92M	35.2±0.5	35.4±0.4	32.7±0.4	31.3±0.5	26.0±0.4	19.6±0.3
E6A/R92A	28.4±0.3	28.5±0.4	26.2±0.4	25.1±0.3	19.9±0.3	15.3±0.3
E6A/R92M	29.7±0.3	30.0±0.4	27.7±0.4	26.1±0.3	21.4±0.3	16.0±0.3
E62A/K46A	27.0±0.3	27.0±0.3	24.0±0.3	21.2±0.3	14.5±0.3	8.2±0.5
E62A/K46M	27.8±0.3	27.8±0.3	25.1±0.3	23.2±0.3	18.4±0.2	12.9±0.2
E90A/R92A	32.4±0.4	32.0±0.5	29.7±0.5	29.4±0.5	22.9±0.4	18.3±0.3
E90A/R92M	33.1±0.3	33.3±0.4	30.3±0.4	29.7±0.4	24.7±0.3	19.8±0.3

### Salt-bridges are stabilizing and their interaction energies are insensitive to temperature changes

Next we investigated the temperature dependency of the pair-wise interaction energy. The measurement of ΔG_u_ was extended to 308, 318, 328, 338 and 348 K (Table 1). The values of ΔΔG_int_ were determined accordingly and summarized in Table S2 and Figure S2. Within each double-mutant cycle, there is no significant difference among the ΔΔG_int_ values obtained at different temperatures, and between those derived from R/K→A cycles and from R/K→M cycles. On the other hand, the ΔΔG_int_ values for different pairs of charge residues were significantly different from each other. The average values of ΔΔG_int_ for the salt-bridges E6/R92 and E62/K46 were 2.3±0.3 and 3.9±0.3, respectively, while the value for the control pair was 0.6±0.3 kJ mol^−1^. Our results suggest that the salt-bridges E6/R92 and E62/K46 are stabilizing, and the pair-wise interaction energy appears to be independent of temperatures.

### ΔC_p_ is reduced by pair-wise interaction of salt-bridges

**Figure 2:**
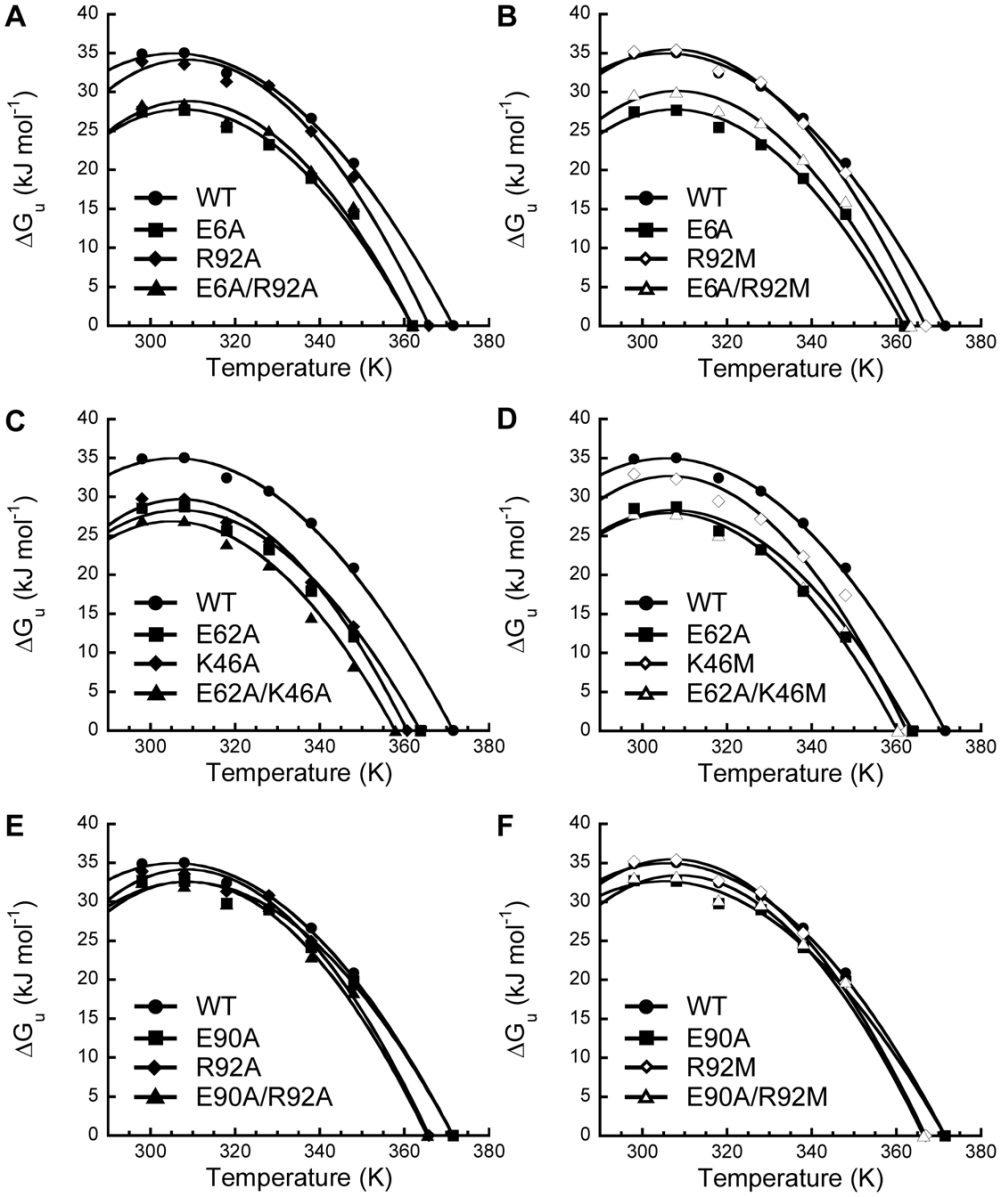
The protein stability curves of *T. celer* L30e and its variants. Values of ΔG_u_ at 298–348 K were obtained by urea-induced denaturation experiments for the variants of L30e in the double-mutant cycles (A) E6A/R92A, (B) E6A/R92M, (C) E62A/K46A, (D) E62A/K46M, (E) E90A/R92A, and (F) E90A/R92M. Values of ΔG_u_ for the wild-type L30e are shown in circles, E→A variants in squares, R/K→A/M variants in diamonds, and the doubly-substituted variants in triangles. Values of ΔG_u_ together with T_m_ were fitted to the Gibbs-Helmholtz equation to obtain values of ΔC_p_.

Values of ΔG_u_ at temperatures 298–348 K and their T_m_ values were fitted to the Gibbs-Helmholtz equation to obtain the values of ΔC_p_ (Figure 2 and Table 2). Compared to the wild-type value of 5.3 kJ mol^−1^ K^−1^, ΔC_p_ were increased to 5.7–6.8 kJ mol^−1^ K^−1^ for substitutions (E6A, E62A, K46A/M, R92A/M) that break a salt-bridge interaction. On the other hand, for the E90A substitution that did not break any salt-bridge, there was no significant change in the value of ΔC_p_ (Table 2). These results suggest that single substitutions that break a salt-bridge would increase the values of ΔC_p_.

**Table 2:** ΔC_p_ (kJ mol^−1^ K^−1^) of *T. celer* L30e and its variants.

Protein sample	Gibbs-Helmholtz analysis	Kirchhoff analysis
Wild-type	5.3±0.4	3.9±0.2
E6A	6.1±0.3	4.6±0.2
K46A	6.8±0.2	4.8±0.3
K46M	6.8±0.4	ND
E62A	5.7±0.2	4.6±0.2
E90A	5.4±0.2	4.0±0.1
R92A	6.8±0.3	4.9±0.2
R92M	6.5±0.3	ND
E6A/R92A	6.7±0.2	4.8±0.1
E6A/R92M	6.5±0.3	ND
E62A/K46A	6.2±0.2	4.6±0.2
E62A/K46M	6.3±0.3	ND
E90A/R92A	6.6±0.2	4.8±0.2
E90A/R92M	6.5±0.3	ND

**Figure 3:**
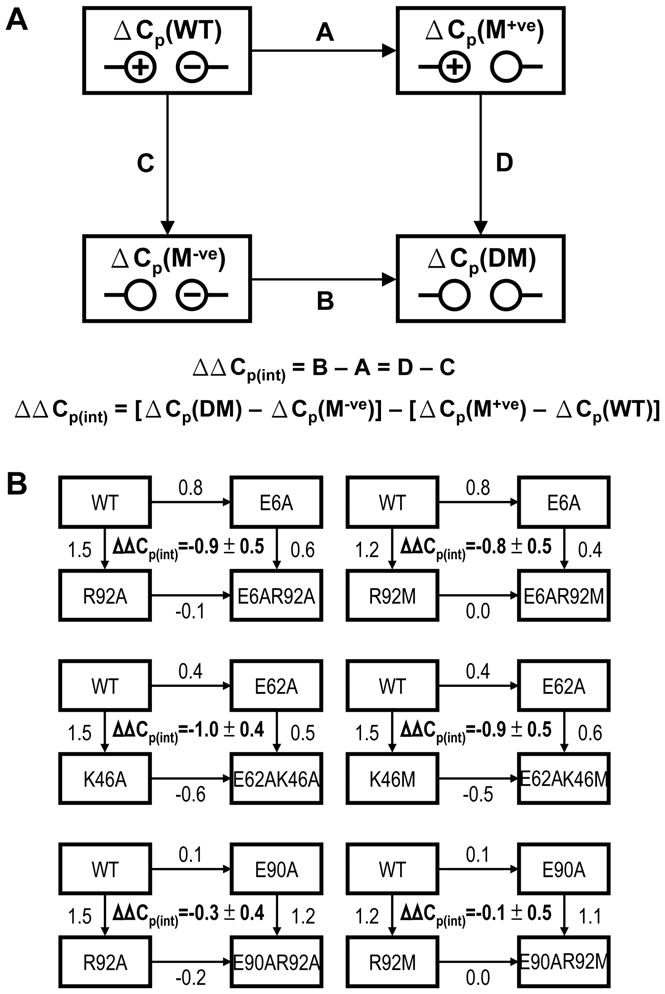
Determination of ΔΔC_p(int)_ by double-mutant cycle analysis. The scheme shown in panel (A) is in analogy to that used to calculate ΔΔG_int_ in Figure S1. (B) ΔΔC_p(int)_ for all six double-mutant cycles analyzed. The substitutions are indicated inside the boxes. The values of ΔΔC_p_ for processes A–D were shown along the arrows, and the values of ΔΔC_p(int)_ were shown in the middle of the cycles. All values are in kJ mol^−1^ K^−1^.

To address the question if the pair-wise interaction of salt-bridges affects the values of ΔC_p_, we determined the ΔΔC_p(int)_ by double-mutant cycle in an analogy to the determination of the ΔΔG_int_ (Figure 3A). Take the double-mutant cycle of E6A/R92A as an example (Figure 3B). Removal of a negative charge by E6A substitution from the wild-type L30e resulted in an increase of ΔC_p_ for 0.8 kJ mol^−1^ K^−1^. On the other hand, the difference in ΔC_p_ between R92A and E6A/R92A was only −0.1 kJ mol^−1^ K^−1^. These data suggest that the two substitutions are not independent, and the pair-wise interaction between E6A and R92A affects the ΔC_p_.

Similar to the argument for the determination of ΔΔG_int_, we have ΔΔC_p(int)_ = [ΔC_p_(DM)−ΔC_p_(M^−ve^)]−[ΔC_p_(M^+ve^)−ΔC_p_(WT)] (Figure 3A). The values of ΔΔC_p(int)_ for the six double-mutant cycles were determined by the double-mutant cycle (Table 3 and Figure 3B). The values of ΔΔC_p(int)_ for the control cycle, E90A/R92A(M), were close to zero (−0.1 to −0.3 kJ mol^−1^ K^−1^). In contrast, for the cycles, E6A/R92A(M) and E62A/K46A(M), that involves breakage of a salt-bridge, values of ΔΔC_p(int)_ were from −0.8 to −1.0 kJ mol^−1^ K^−1^. The negative values of ΔΔC_p(int)_ strongly suggest that the pair-wise interaction of salt-bridges reduces the ΔC_p_.

**Table 3:** ΔΔC_p(int)_ (kJ mol^−1^ K^−1^) determined by double-mutant cycles.

Double-mutant Cycles	Gibbs-Helmholtz analysis	Kirchhoff analysis
E6A/R92A	−0.9±0.6	−0.8±0.4
E6A/R92M	−0.8±0.6	ND
E62A/K46A	−1.0±0.5	−0.9±0.5
E62A/K46M	−0.9±0.7	ND
E90A/R92A	−0.3±0.6	−0.2±0.4
E90A/R92M	−0.1±0.6	ND

**Figure 4:**
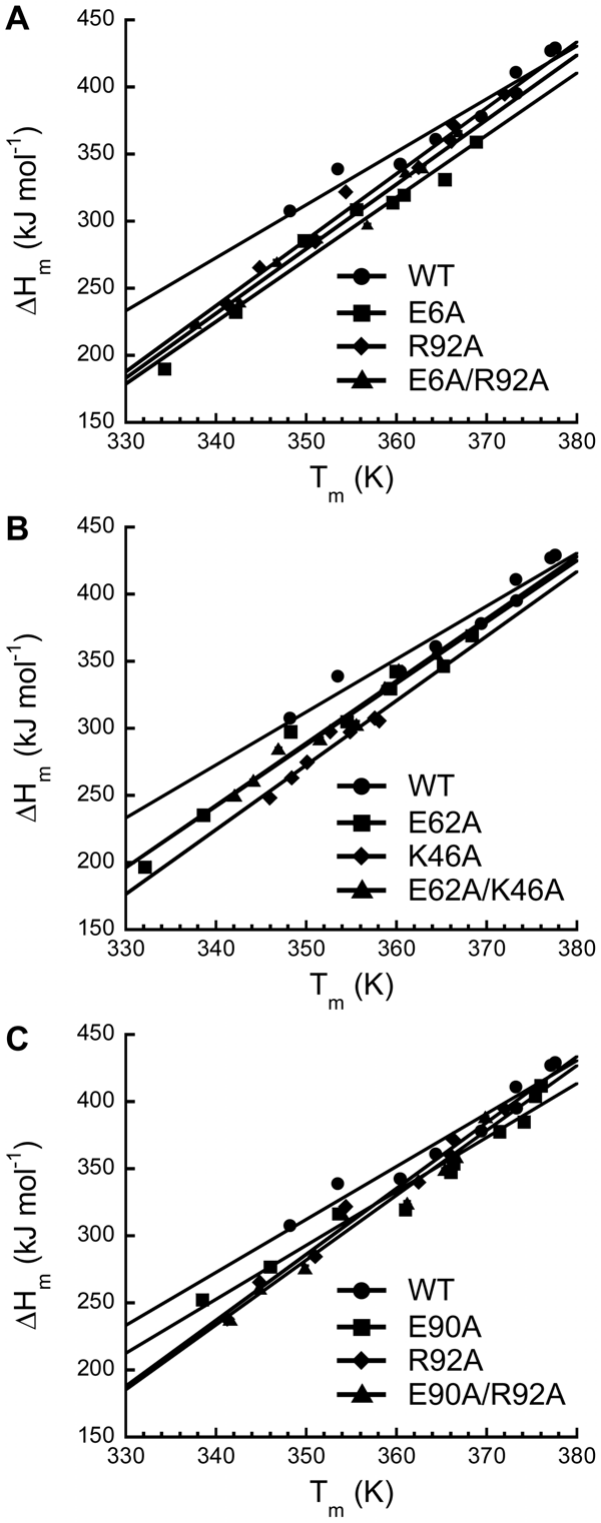
Determination of ΔC_p_ by the Kirchhoff analysis. ΔH_m_ and T_m_ were obtained at pH 2.6–6.0 by thermal denaturation for the variants of L30e in the double-mutant cycles (A) E6A/R92A, (B) E62A/K46A, and (C) E90A/R92A. Values of ΔH_m_ were plotted as a function of T_m_ for *T. celer* L30e and its variants. ΔC_p_ was obtained by the slope of the plot.

To further confirm the hypothesis that the pair-wise interaction of salt-bridge contributes to the reduction of ΔC_p_, we have determined the values of ΔC_p_ independently by the Kirchhoff analysis [35]–[37]. Values of T_m_ and ΔH_m_ at pH 2.5–6.0 for L30e and its variants were obtained by thermal denaturation. ΔC_p_ values for wild-type and variant L30e were derived from the slope of the ΔH_m_ vs. T_m_ plot (Figure 4), and summarized in Table 2. The ΔC_p_ value for wild-type L30e was 3.9±0.2 kJ mol^−1^ K^−1^. For substitutions (E6A, K46A, E62A, R92A) that break a salt-bridge, the ΔC_p_ values were increased to 4.6–4.9 kJ mol^−1^ K^−1^ (Table 2). On the other hand, for E90A substitution that did not break any salt-bridge, the ΔC_p_ value was 4.0±0.1 kJ mol^−1^ K^−1^, which was similar to that of wild-type L30e.

We noticed that ΔC_p_ values obtained using the Kirchhoff analysis based on thermal denaturation data were smaller than those using Gibbs-Helmholtz analysis based on chemical-induced denaturation experiments. This observation is consistent with our previous report, in which we pointed out that the systematic differences in ΔC_p_ values were probably due to the thermal denatured state having more residual structures than the chemical-induced denatured state [12].

Regardless of the systematic differences in ΔC_p_ values, the values of ΔΔC_p(int)_ determined by the Kirchhoff analysis were in striking agreement with those obtained by the Gibbs-Helmholtz analysis (Table 3). For the double-mutant cycles involving the breakage of a salt-bridge, the values of ΔΔC_p(int)_ were −0.8±0.4 and −0.9±0.5 kJ mol^−1^ K^−1^ for E6A/R92A and E62A/K46A, respectively. In contrast, the ΔΔC_p(int)_ was close to zero for the control cycle E90A/R92A (−0.2±0.4 kJ mol^−1^ K^−1^). Taken together, our results suggest that the pair-wise interaction of salt-bridge reduces the ΔC_p_ by ca. 0.8–1.0 kJ mol^−1^ K^−1^.

### No major structural changes were observed in the double charge-to-Ala variants

The crystal structures of the E6A/R92A, K46A/E62A, and E90A/R92A variants were determined at resolution ranging from 1.8 to 2.0 Å (Table S3). The structures of all these variants can be superimposable with the wild-type structures (Figure S3). The root-mean-square deviations (r.m.s.d.) between C^α^ atoms of the wild-type L30e and its variants were <0.5 Å (Table S3), suggesting there were no major structural change in these variants.

## Discussion

Whether salt-bridge contributes to protein stability is controversial, and is probably context dependent [23], [30], [38]–[42]. Elcock proposed that salt-bridge should be more stabilizing at high temperatures because the unfavorable desolvation penalty [43]–[45] and the entropic cost of fixing two charged side-chains [33], [46], [47] would decrease with temperatures [48]. Here, we used the double-mutant-cycle approach to study how salt-bridge contributes to the thermostability of proteins. The two salt-bridges, E6/R92 and E62/K46, stabilizes the protein by ∼2–5 kJ mol^−1^ (Figure S2 and Table S2). That values of ΔΔG_int_ for R/K→A and R/K→M cycles were similar suggests that the stabilization is mainly due to the charge-charge interaction, rather than hydrophobic interaction, between the salt-bridging residues. We showed that the pair-wise interaction energy, ΔΔG_int_, is insensitive to temperature changes (Figure S2). This observation is consistent with a previous study by Ge and co-workers [26], which showed that the pair-wise interaction energies of salt-bridges in a hyperthermophilic protein Ssh10b at 298 and 353 K were similar. Since the free energy of unfolding is decreasing with temperatures, the more-or-less constant stabilizing effect of salt-bridges should contribute more in proportion to the overall protein stability at high temperatures.

We further demonstrated unambiguously that the stabilizing salt-bridges reduce the heat capacity change of unfolding (ΔC_p_). We showed that single-substitutions that break a salt-bridge increased the ΔC_p_ value. This observation is consistent with our previous report in that removal of favorable electrostatic interactions by single charge-to-neutral substitutions increases the ΔC_p_ [12]. Using the double-mutant-cycle approach, we determined the values of ΔΔC_p(int)_, which estimates how much the pair-wise interaction between the salt-bridging residues contributes to the heat capacity change of unfolding. For the double-mutant cycles that break a salt-bridge (i.e. E6/R92 and E62/K46), negative values of ΔΔC_p(int)_ suggest that the pair-wise interaction of the salt-bridges reduces the ΔC_p_ by 0.8 to 1.0 kJ mol^−1^ K^−1^.

Using guanidine-induced denaturation and Gibbs-Helmholtz analysis, we have previously showed that the ΔC_p_ for the mesophilic L30e from yeast (10.5 kJ mol^−1^ K^−1^) was much larger than that for the thermophilic *T. celer* L30e (5.3 kJ mol^−1^ K^−1^) [12]. It is in fact a common observation that thermophilic proteins tend to have smaller values of ΔC_p_ than their mesophilic homologues [4]–[8], [10], [11]. Here, we demonstrated by double-mutant cycle that the pair-wise interaction between the salt-bridging residues reduces the ΔC_p_, which provide a structural basis of why thermophilic proteins have smaller values of ΔC_p_. This conclusion is consistent with our previous observation that removal of favorable charge-charge interactions by single substitutions resulted in increases in ΔC_p_ [12]. Using a simple spherical model, Zhou predicted that favorable interaction between two oppositely charge residues should decrease ΔC_p_ [49]. Our experimental results provide unambiguous evidence supporting the conclusion that stabilizing salt-bridge reduces the ΔC_p_.

The structural basis of why thermophilic proteins have smaller values of ΔC_p_ is controversial. It has been well documented that ΔC_p_ correlates well with the changes in solvent accessible surface area (ΔASA) upon unfolding [50]–[52]. As we have pointed out previously, due to the similarity in their native conformation, homologous proteins tend to bury similar amount of ASA upon folding assuming the denatured states are random coil [12]. To explain the differences in ΔC_p_ between thermophilic and mesophilic proteins, it has been proposed that thermophilic proteins may have more residual structures in their denatured states so that the ΔASA would be smaller than that calculated for a random-coil [53], [54]. However, it is uncertain if the differences in residual structures, if any, can explain the large differences in ΔC_p_ observed. Moreover, Zhou pointed out that the presence of more residual structures may increase the free energy of the denatured state and destabilize the protein, which is counter-intuitive to the fact that thermophilic proteins are more stable than their mesophilic homologs [49]. Apparently, the correlation of ΔC_p_ to ΔASA fails to account for the large differences in ΔC_p_ commonly observed for thermophilic and mesophilic pairs of homologous proteins [8], [9], suggesting that factors other than the hydration effect may also contribute to ΔC_p_.

Our results showed that the ΔH_m_ for the wild-type protein was slightly higher than that for the variants (Figure 4). Under this condition, having a smaller ΔC_p_ always enhances protein thermostability by up-shifting and broadening the protein stability curve. Figure S4 simulates the shape of the protein stability curve of two hypothetical proteins with ΔC_p_ values of 5.3 and 7.3 kJ mol^−1^ K^−1^. The simulation shows that a decrease of ΔC_p_ by 2 kJ mol^−1^ K^−1^ shifts the protein stability upward, and increases its maximum stability by ∼10 kJ mol^−1^. It also broadens the protein stability curve so that the protein remains stable at a wilder range of temperatures. Our previous study also showed *T. celer* L30e has an up-shifted and broadened protein stability curve when compare with that of the mesophilic yeast homologues [12].

In a survey of 26 protein families where thermodynamics data were available for both mesophilic and thermophilic homologs, Razvi and co-workers found that most protein enhances their thermostability by up-shifting and broadening of the protein stability curves [3]. Since thermophilic proteins tend to have more salt-bridges than their mesophilic homologs [14]–[16], our observation that salt-bridge reduces ΔC_p_ may provide a general mechanism for enhancing thermostability - thermophilic proteins have more stabilizing salt-bridges that reduce the ΔC_p_, leading to the up-shifting and broadening of the protein stability curve.

## Materials and Methods

### Site-directed mutagenesis

All site-directed mutagenesis were performed by a two-stage PCR procedure modified from the QuikChange site-directed mutagenesis protocol using the mutagenic primers listed in Table S1 [55]. Wild-type *T. celer* L30e cloned in expression vector pET3d (Novagen) was used as the template in all polymerase reactions. Mutations introduced were confirmed by DNA sequencing.

### Protein expression and purification

All protein samples were expressed and purified as described [21], [56].

### Thermal-induced denaturation

20 µM protein samples were dialyzed in 10 mM sodium acetate buffer at pH 5.4 for 16 hours before circular dichroism (CD) measurement. After degassing thoroughly, all protein samples were heated in a securely stoppered 1 mm path-length cuvette from 298 K to 383 K at a heating rate of 1 K min^−1^. The thermal denaturation was then monitored by molar ellipticity at 222 nm using a JASCO J810 spectropolarimeter equipped with a peltier-type temperature control unit.

The melting temperature (T_m_) and enthalpy of unfolding ΔH_m_ were obtained by fitting the thermal denaturation curve to a two-state model (Figure S5):
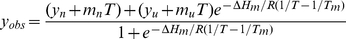
where y_obs_ is the observed molar ellipticity at 222 nm; y_n_ and m_n_ are the y-intercept and slope of the pre-transition baseline; y_u_ and m_u_ are the y-intercept and slope of the post-transition baseline; R is the gas constant; T is the temperature in Kelvin.

### Urea-induced denaturation

20 µM protein samples were equilibrated with 0 M–10.2 M urea in 10 mM sodium acetate buffer at pH 5.4 for 30 minutes before CD measurement. Concentration of urea was determined from refractive index measurements [57] using Leica AR200 refractometer. The urea-induced denaturation was monitored by molar ellipticity at 222 nm using a JASCO J810 spectropolarimeter equipped with a peltier-type temperature control unit. The urea-induced denaturation was analyzed by a two-state model [58] (Figure S6):
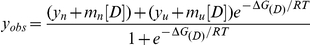
where y_obs_ is the observed molar ellipticity at 222 nm; y_n_ and m_n_ are the y-intercept and slope of the pre-transition baseline; y_u_ and m_u_ are the y-intercept and slope of the post-transition baseline; R is the gas constant; T is the temperature in Kelvin; [D] is the concentration of urea; ΔG_(D)_ is the free energy change of unfolding at [D]. The free energy change of unfolding without denaturant, ΔG_u_, was obtained by linear extrapolation model [58]: ΔG_(D)_ = ΔG_u_−m[D], using the average m-value approach [59]. ΔG_u_ at 298 K, 308 K, 318 K, 328 K, 338 K, and 348 K were measured for *T. celer* L30e and its mutants.

### Estimation of ΔC_p_ by Gibbs-Helmholtz analysis

ΔG_u_ at temperatures from 298 K to 348 K and T_m_ were fitted to the Gibbs-Helmholtz equation to obtain the values of ΔC_p_. For variants of L30e (E6A, K46M, E6AR92A, and E6AR92M) that exhibited irreversible thermal denaturation, values of apparent T_m_ were used. The program PRISM (GraphPad Software, La Jolla, USA) was used to estimate the errors in ΔC_p_ due to the uncertainty in ΔG_u_.

### Estimation of ΔC_p_ by Kirchhoff analysis

Thermal-induced denaturation curves were measured for protein samples of *T. celer* L30e in 10 mM sodium citrate/phosphate buffer at pH 2.5 to 6.0. T_m_ and ΔH_m_ were obtained from by fitting the data to the two-state model described above. ΔC_p_ values were then obtained from the slope of the ΔH_m_ vs. T_m_ plot. Only the data obtained from reversible thermal denaturation were included in the Kirchhoff analysis.

### Crystal structure determination

Crystals of L30e variants were grown by sitting-drop-vapor-diffusion method at 289 K. 2 µl of 10 mg ml^−1^ protein sample was mixed with 2 µl of precipitant solution (Table S3). Datasets were acquired and collected at 100 K using an in-house rotating anode X-ray source. The diffraction data were processed, merged, scaled, and reduced with programs (MOSFLM, SCALA, TRUNCATE) from the CCP4 suite [60]. The structures were solved by molecular replacement using PHENIX with the wild-type *T. celer* L30e crystal structure (PDB code: 1H7M) as the search model. The structures were refined using PHENIX [61], and were validated using WHATCHECK [62] and MOLPROBITY [63], [64].

## Supporting Information

Figure S1 Coupling energies (ΔΔG_int_) were determined by double-mutant cycles. (A) The scheme explaining how ΔΔG_int_ are calculated from values of ΔG_u_ for wild-type (WT), single-mutants (M^+ve^ and M^−ve^), and double-mutant (DM) by the double-mutant cycle analysis. (B) ΔΔG_int_ for all six double-mutant cycles analyzed. The substitutions are indicated inside the boxes. The values of ΔΔG_u_ for processes A–D were shown along the arrows, and the values of ΔΔG_int_ were shown in the middle of the cycles. All values are in kJ mol^−1^.
(PDF)

Figure S2 Temperature dependency of the coupling energy. Values of ΔΔG_int_ derived from double-mutant cycles (A) E6A/R92A(M) (circles), (B) E62A/K46A(M) (squares), and (C) E90A/R92A(M) (diamonds) at temperatures 298 K to 348 K are shown. Values of ΔΔG_int_ derived from the R/K→A cycles are represented by filled symbols, and those from the R/K→M cycles by open symbols.
(PDF)

Figure S3 Crystal structures of variants of *T. celer* L30e. Crystal structures of E6A/R92A (red), E62A/K46A (green), and E90A/R92A (blue) are superimposable to the wild-type *T. celer* L30e (black).
(PDF)

Figure S4 Reduced ΔC_p_ up-shifts and broadens the protein stability curve. The protein stability curve of a hypothetical protein with ΔC_p_ = 7.3 kJ mol^−1^ K^−1^, T_m_ = 356 K, ΔH_m_ = 382 kJ mol^−1^ was simulated using the Gibbs-Helmholtz equation (dashed line). Keeping ΔH_m_ and T_s_ (temperature for maximum stability) constant, the protein stability curve with a reduced value of ΔC_p_ = 5.3 kJ mol^−1^ K^−1^ was simulated as the solid line.
(PDF)

Figure S5 Thermal denaturation of wild-type *T. celer* L30e at different pH. The thermal denaturation curves of wild-type *T. celer* L30e in 10 mM citrate/phosphate buffer at pH ranging from 2.5 to 6.0 were shown.
(PDF)

Figure S6 Urea-induced denaturation of wild-type *T. celer* L30e at different temperatures. The 52-point urea-induced denaturation curves of wild-type *T. celer* L30e in 10 mM sodium acetate buffer, pH 5.4 at temperatures ranging from 298 K to 348 K were shown.
(PDF)

Table S1 Oligonucleotide primers used in the mutagenesis.
(DOC)

Table S2 ΔΔG_int_ at 298–348 K determined by double-mutant cycles.
(DOC)

Table S3 Statistics for crystal structure determination of E6A/R92A, E62A/K46A, E90A/R92A.
(DOC)
