# Supplementary material for: Stabilizing Salt-Bridge Enhances Protein Thermostability by Reducing the Heat Capacity Change of Unfolding
Source: PLoS One. 2011 Jun 24;6(6):e21624. doi: 10.1371/journal.pone.0021624 (PMC3123365; doi:10.1371/journal.pone.0021624)
Supplement: Figure S1 — Coupling energies (ΔΔGint) were determined by double-mutant cycles. (A) The scheme explaining how ΔΔGint are calculated from values of ΔGu for wild-type (WT), single-mutants (M+ve and M−ve), and double-mutant (DM) by the double-mutant cycle analysis. (B) ΔΔGint for all six double-mutant cycles analyzed. The substitutions are indicated inside the boxes. The values of ΔΔGu for processes A–D were shown along the arrows, and the values of ΔΔGint were shown in the middle of the cycles. All values are in kJ mol−1. (PDF) [file pone.0021624.s001.pdf]

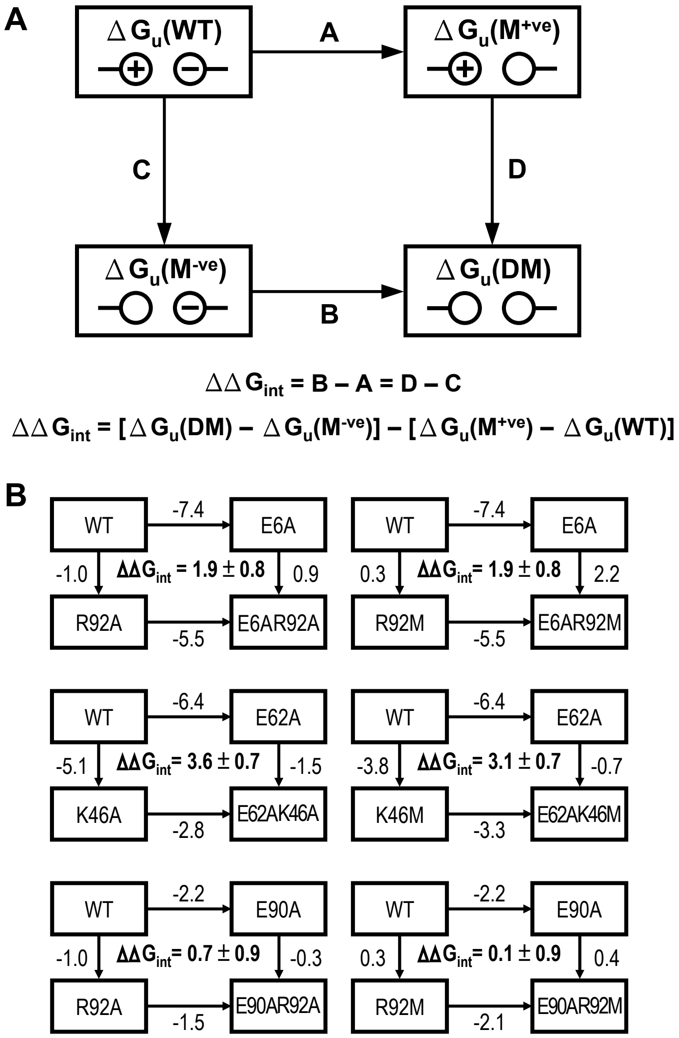

Figure S1. Coupling energies ($\Delta\Delta G_{int}$) were determined by double-mutant cycles. (A) The scheme explaining how $\Delta\Delta G_{int}$ are calculated from values of ΔG_u for wild-type (WT), single-mutants (M^{+ve} and M^{-ve}), and double-mutant (DM) by the double-mutant cycle analysis. (B) $\Delta\Delta G_{int}$ for all six double-mutant cycles analyzed. The substitutions are indicated inside the boxes. The values of $\Delta\Delta G_u$ for processes A – D were shown along the arrows, and the values of $\Delta\Delta G_{int}$ were shown in the middle of the cycles. All values are in kJ mol^{-1}.
