# Supplementary material for: Stabilizing Salt-Bridge Enhances Protein Thermostability by Reducing the Heat Capacity Change of Unfolding
Source: PLoS One. 2011 Jun 24;6(6):e21624. doi: 10.1371/journal.pone.0021624 (PMC3123365; doi:10.1371/journal.pone.0021624)
Supplement: Figure S2 — Temperature dependency of the coupling energy. Values of ΔΔGint derived from double-mutant cycles (A) E6A/R92A(M) (circles), (B) E62A/K46A(M) (squares), and (C) E90A/R92A(M) (diamonds) at temperatures 298 K to 348 K are shown. Values of ΔΔGint derived from the R/K→A cycles are represented by filled symbols, and those from the R/K→M cycles by open symbols. (PDF) [file pone.0021624.s002.pdf]

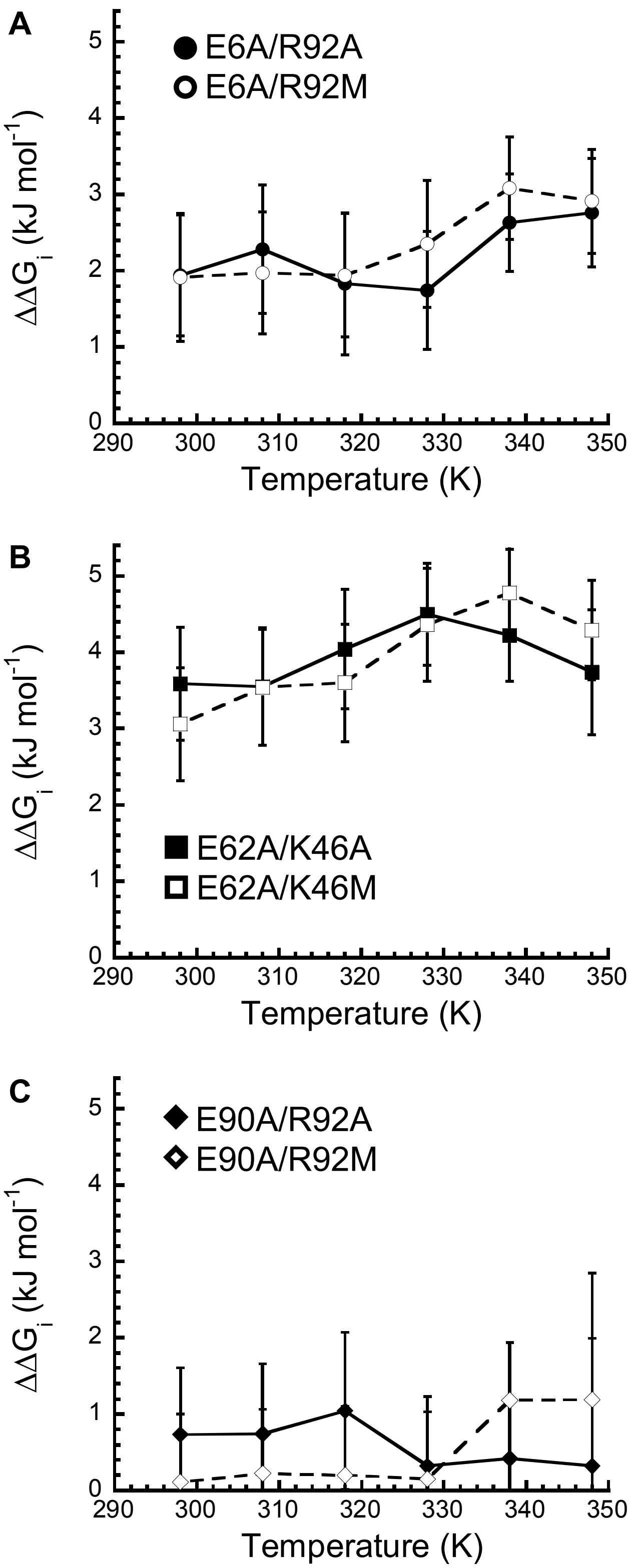

Figure S2. Temperature dependency of the coupling energy. Values of $\Delta\Delta G_{int}$ derived from double-mutant cycles (A) E6A/R92A(M) (circles), (B) E62A/K46A(M) (squares), and (C) E90A/R92A(M) (diamonds) at temperatures 298 K to 348 K are shown. Values of $\Delta\Delta G_{int}$ derived from the R/K→A cycles are represented by filled symbols, and those from the R/K→M cycles by open symbols.
