# Supplementary material for: Stabilizing Salt-Bridge Enhances Protein Thermostability by Reducing the Heat Capacity Change of Unfolding
Source: PLoS One. 2011 Jun 24;6(6):e21624. doi: 10.1371/journal.pone.0021624 (PMC3123365; doi:10.1371/journal.pone.0021624)
Supplement: Figure S3 — Crystal structures of variants of T. celer L30e. Crystal structures of E6A/R92A (red), E62A/K46A (green), and E90A/R92A (blue) are superimposable to the wild-type T. celer L30e (black). (PDF) [file pone.0021624.s003.pdf]

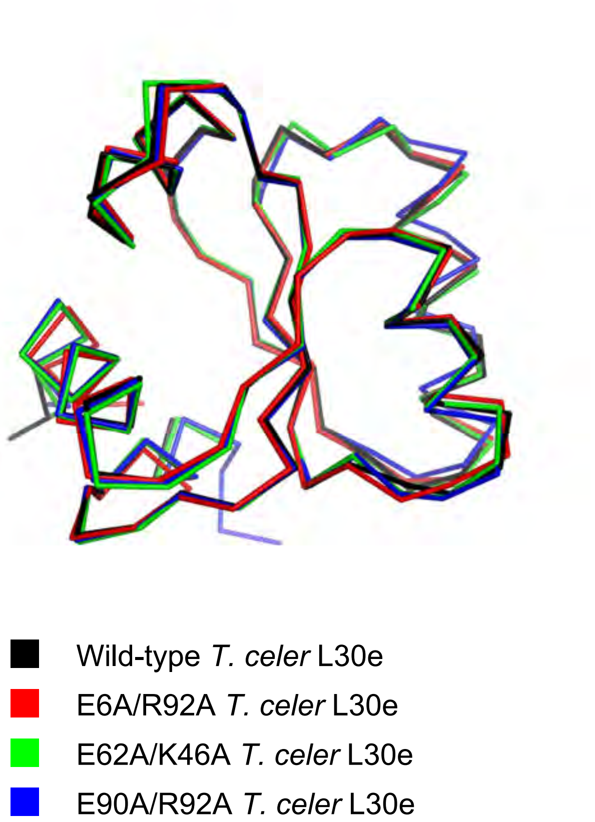

Figure S3. Crystal structures of variants of *T. celer* L30e. Crystal structures of E6A/R92A (red), E62A/K46A (green), and E90A/R92A (blue) are superimposable to the wild-type *T. celer* L30e (black).
