# Supplementary material for: Stabilizing Salt-Bridge Enhances Protein Thermostability by Reducing the Heat Capacity Change of Unfolding
Source: PLoS One. 2011 Jun 24;6(6):e21624. doi: 10.1371/journal.pone.0021624 (PMC3123365; doi:10.1371/journal.pone.0021624)
Supplement: Figure S4 — Reduced ΔCp up-shifts and broadens the protein stability curve. The protein stability curve of a hypothetical protein with ΔCp = 7.3 kJ mol−1 K−1, Tm = 356 K, ΔHm = 382 kJ mol−1 was simulated using the Gibbs-Helmholtz equation (dashed line). Keeping ΔHm and Ts (temperature for maximum stability) constant, the protein stability curve with a reduced value of ΔCp = 5.3 kJ mol−1 K−1 was simulated as the solid line. (PDF) [file pone.0021624.s004.pdf]

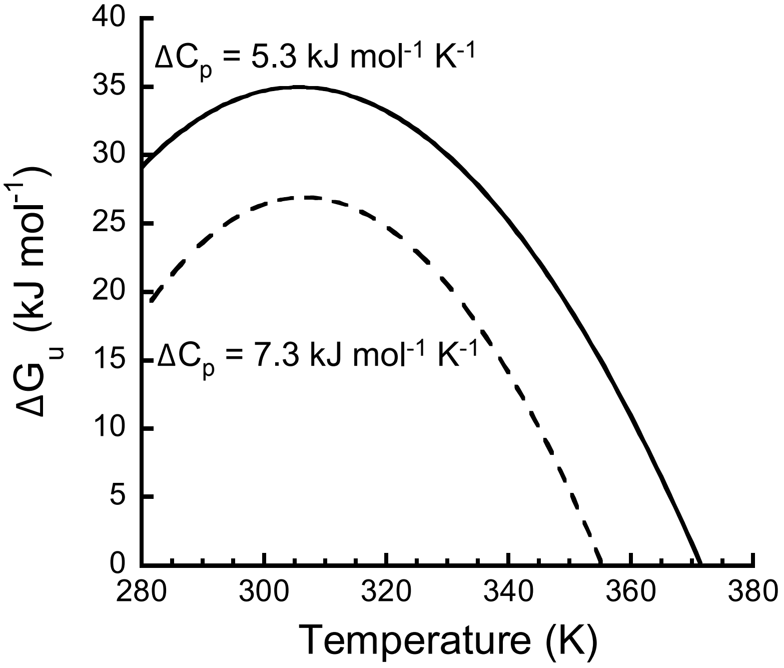

Figure S4. Reduced ΔC_p up-shifts and broadens the protein stability curve. The protein stability curve of a hypothetical protein with ΔC_p= 7.3 kJ mol^{-1} K^{-1}, T_m= 356 K, ΔH_m= 382 kJ mol^{-1} was simulated using the Gibbs-Helmholtz equation (dashed line). Keeping ΔH_m and T_s (temperature for maximum stability) constant, the protein stability curve with a reduced value of ΔC_p=5.3 kJ mol^{-1} K^{-1} was simulated as the solid line.
