# Supplementary material for: Stabilizing Salt-Bridge Enhances Protein Thermostability by Reducing the Heat Capacity Change of Unfolding
Source: PLoS One. 2011 Jun 24;6(6):e21624. doi: 10.1371/journal.pone.0021624 (PMC3123365; doi:10.1371/journal.pone.0021624)
Supplement: Figure S5 — Thermal denaturation of wild-type T. celer L30e at different pH. The thermal denaturation curves of wild-type T. celer L30e in 10 mM citrate/phosphate buffer at pH ranging from 2.5 to 6.0 were shown. (PDF) [file pone.0021624.s005.pdf]

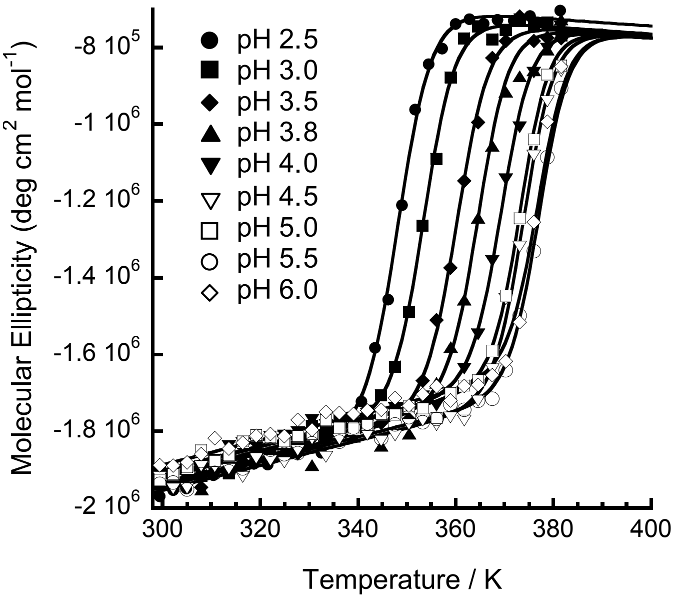

Figure S5. Thermal denaturation of wild-type T. celer L30e at different pH. The thermal-induced denaturation curves of wild-type T. celer L30e in 10 mM citrate/phosphate buffer at pH ranging from 2.5 to 6.0 were shown.
