# Supplementary material for: Stabilizing Salt-Bridge Enhances Protein Thermostability by Reducing the Heat Capacity Change of Unfolding
Source: PLoS One. 2011 Jun 24;6(6):e21624. doi: 10.1371/journal.pone.0021624 (PMC3123365; doi:10.1371/journal.pone.0021624)
Supplement: Figure S6 — Urea-induced denaturation of wild-type T. celer L30e at different temperatures. The 52-point urea-induced denaturation curves of wild-type T. celer L30e in 10 mM sodium acetate buffer, pH 5.4 at temperatures ranging from 298 K to 348 K were shown. (PDF) [file pone.0021624.s006.pdf]

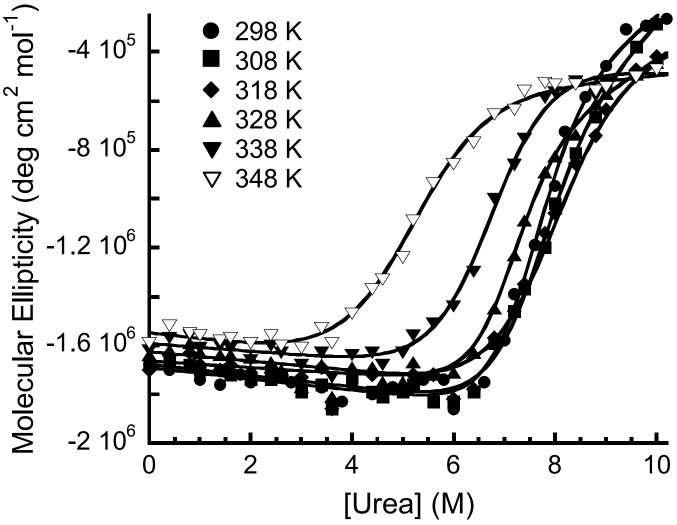

Figure S6. Urea-induced denaturation of wild-type T. celer L30e at different temperatures. The 52-point urea-induced denaturation curves of wild-type T. celer L30e in 10 mM sodium acetate buffer, pH 5.4 at temperatures ranging from 298 K to 348 K were shown.
