# Supplementary material for: Stabilizing Salt-Bridge Enhances Protein Thermostability by Reducing the Heat Capacity Change of Unfolding
Source: PLoS One. 2011 Jun 24;6(6):e21624. doi: 10.1371/journal.pone.0021624 (PMC3123365; doi:10.1371/journal.pone.0021624)
Supplement: Table S1 — Oligonucleotide primers used in the mutagenesis. (DOC) [file pone.0021624.s007.doc]

**Table S1.** Oligonucleotide primers used in the mutagenesis

| **Variants** | **Primers** | **Primers sequences** |
| --- | --- | --- |
| E6A | E6A_F | GTTGATTTTGCTTTCGCGCTCCGTAAGGCTCAG |
|  | FLANK_R | TCACTCTTTACCGCCCAACGCCAGAATACGAGA |
| K46A | K46A_F | GCGCGCCCTGATATTGCGGAAGACATCGAATAC |
|  | FLANK_R | TCACTCTTTACCGCCCAACGCCAGAATACGAGA |
| K46M | K46M_F | GCGCGCCCTGATATTATGGAAGACATCGAATAC |
|  | FLANK_R | TCACTCTTTACCGCCCAACGCCAGAATACGAGA |
| E62A | FLANK_F | ATGGTTGATTTTGCTTTCGAACTCCGTAAGGCT |
|  | E62A_R | GGAGGTGCCCTCGAACGCATACACTGGAATACC |
| E90A | FLANK_F | ATGGTTGATTTTGCTTTCGAACTCCGTAAGGCT |
|  | E90A_R | CGCCAGAATACGAGACGCACCCGGGTCCACGAC |
| R92A | FLANK_F | ATGGTTGATTTTGCTTTCGAACTCCGTAAGGCT |
|  | R92A_R | GCCCAACGCCAGAATCGCAGATTCACCCGGGTC |
| R92M | FLANK_F | ATGGTTGATTTTGCTTTCGAACTCCGTAAGGCT |
|  | R92M_R | GCCCAACGCCAGAATCATAGATTCACCCGGGTC |
| E6A/R92A | E6A_F | GTTGATTTTGCTTTCGCGCTCCGTAAGGCTCAG |
|  | R92A_R | GCCCAACGCCAGAATCGCAGATTCACCCGGGTC |
| E6A/R92M | E6A_F | GTTGATTTTGCTTTCGCGCTCCGTAAGGCTCAG |
|  | R92M_R | GCCCAACGCCAGAATCATAGATTCACCCGGGTC |
| E62A/K46A | K46A_F | GCGCGCCCTGATATTGCGGAAGACATCGAATAC |
|  | E62A_R | GGAGGTGCCCTCGAACGCATACACTGGAATACC |
| E62A/K46M | K46M_F | GCGCGCCCTGATATTATGGAAGACATCGAATAC |
|  | E62A_R | GGAGGTGCCCTCGAACGCATACACTGGAATACC |
| E90A/R92A | FLANK_F | ATGGTTGATTTTGCTTTCGAACTCCGTAAGGCT |
|  | E90AR92A_R | GCCCAACGCCAGAATCGCAGACGCACCCGGGTCCACGAC |
| E90A/R92M | FLANK_F | ATGGTTGATTTTGCTTTCGAACTCCGTAAGGCT |
|  | E90AR92M_R | GCCCAACGCCAGAATCATAGACGCACCCGGGTCCACGAC |
| Bases that are underlined are designed mutations in the *T. celer* L30e sequence. | | |
