# Supplementary material for: Stabilizing Salt-Bridge Enhances Protein Thermostability by Reducing the Heat Capacity Change of Unfolding
Source: PLoS One. 2011 Jun 24;6(6):e21624. doi: 10.1371/journal.pone.0021624 (PMC3123365; doi:10.1371/journal.pone.0021624)
Supplement: Table S2 — ΔΔGint at 298–348 K determined by double-mutant cycles. (DOC) [file pone.0021624.s008.doc]

Table S2. ∆∆G_int_ at 298 – 348 K determined by double-mutant cycles

| **Temperature (K)** | **E6A /**  **R92A**  **(kJ mol^-1^)** | **E6A /**  **R92M**  **(kJ mol^-1^)** | **E62A /**  **K46A**  **(kJ mol^-1^)** | **E62A /**  **K46M**  **(kJ mol^-1^)** | **E90A /**  **R92A**  **(kJ mol^-1^)** | **E90A /**  **R92M**  **(kJ mol^-1^)** |
| --- | --- | --- | --- | --- | --- | --- |
| 298 | 1.9 ± 0.8 | 1.9 ± 0.8 | 3.6 ± 0.7 | 3.1 ± 0.7 | 0.7 ± 0.9 | 0.1 ± 0.9 |
| 308 | 2.3 ± 0.8 | 2.0 ± 0.8 | 3.6 ± 0.8 | 3.5 ± 0.8 | 0.7 ± 0.9 | 0.2 ± 0.8 |
| 318 | 1.8 ± 0.9 | 1.9 ± 0.8 | 4.0 ± 0.8 | 3.6 ± 0.8 | 1.0 ± 1.0 | 0.2 ± 0.9 |
| 328 | 1.7 ± 0.8 | 2.4 ± 0.8 | 4.5 ± 0.7 | 4.4 ± 0.7 | 0.3 ± 0.9 | 0.2 ± 0.9 |
| 338 | 2.6 ± 0.6 | 3.1 ± 0.7 | 4.2 ± 0.6 | 4.8 ± 0.6 | 0.4 ± 0.8 | 1.2 ± 0.8 |
| 348 | 2.8 ± 0.7 | 2.9 ± 0.7 | 3.7 ± 0.8 | 4.3 ± 0.7 | 0.3 ± 1.7 | 1.2 ± 1.7 |
