# Supplementary material for: Stabilizing Salt-Bridge Enhances Protein Thermostability by Reducing the Heat Capacity Change of Unfolding
Source: PLoS One. 2011 Jun 24;6(6):e21624. doi: 10.1371/journal.pone.0021624 (PMC3123365; doi:10.1371/journal.pone.0021624)
Supplement: Table S3 — Statistics for crystal structure determination of E6A/R92A, E62A/K46A, E90A/R92A. (DOC) [file pone.0021624.s009.doc]

Table S3. Statistics for crystal structure determination of E6A/R92A, E62A/K46A, E90A/R92A

|  | **E6A/R92A** | **E62A/K46A** | **E90A/R92A** |
| --- | --- | --- | --- |
| **PDB code** | 3RA5 | 3RA6 | 3LFO |
| **Summary of crystallization conditions:** | | | |
|  | 25% PEG 20000,  0.1 M Tris, pH 8.0, 289 K | 5% PEG MME 2000, 0.1 M MES, pH 6.0,  289 K | 18% PEG 3350, 0.1 M sodium citrate, pH 6.0,  289 K |
| **Diffraction data collection statistics:** | | | |
| X-ray source | Cu Kα | Cu Kα | Cu Kα |
| Space group | P32 | P2_1_ | P2_1_ |
| Resolution (Å) | 36.5-1.8  (1.9-1.8) | 32.4-2.0  (2.1-2.0) | 31.7-1.8  (1.9-1.8) |
| Molecules per asymmetric unit | 2 | 1 | 1 |
| Unit cell dimension (Å) | a, 64.2; b, 64.2;  c, 48.4 | a, 23.6; b, 53.9;  c, 34.3 | a, 23.9; b, 53.1;  c, 33.5 |
| Unit cell angles (deg.) | α, 90.0; β, 90.0; γ, 120.0 | α, 90.0; β, 109.2; γ, 90.0 | α, 90.0; β, 108.6; γ, 90.0 |
| Multiplicity | 2.7 (2.7) | 2.7 (2.7) | 3.0 (3.0) |
| Completeness (%) | 100.0 (100.0) | 99.5 (100.0) | 98.1 (96.3) |
| Mean *I*/σ (*I*) | 11.4 (4.9) | 7.9 (4.8) | 14.8 (3.7) |
| R_merge_ (%) | 6.4 (15.3) | 7.7 (17.4) | 7.3 (24.4) |
| Unique reflections | 20739 (3047) | 5520 (791) | 7279 (1034) |
| **Structural refinement statistics:** | | | |
| R-factor / R_free_ (%) | 19.0 / 23.5 | 19.6 / 25.3 | 16.5 / 20.4 |
| *r.m.s.d. from ideal values:* | | | |
| Bond distances (Å) | 0.006 | 0.007 | 0.027 |
| Bond angles (deg.) | 1.014 | 1.008 | 1.940 |
| *Ramachandran pot analysis:* | | | |
| Preferred region (%) | 97.3 | 97.9 | 99.0 |
| Allowed region (%) | 2.7 | 2.1 | 1.0 |
| Outliers (%) | 0.0 | 0.0 | 0.0 |
| C^α^ r.m.s.d. (wild type T. celer L30e) | 0.43 | 0.42 | 0.49 |
| Values in parentheses are for the highest-resolution shell. | | | |
